# An Agent-Based Model of the Local Spread of SARS-CoV-2: Modeling Study

**DOI:** 10.2196/24192

**Published:** 2021-04-06

**Authors:** Alessio Staffini, Akiko Kishi Svensson, Ung-Il Chung, Thomas Svensson

**Affiliations:** 1 Department of Economics and Finance Catholic University of Milan Milan Italy; 2 Project Promotion Department ALBERT Inc Tokyo Japan; 3 Precision Health, Department of Bioengineering Graduate School of Engineering The University of Tokyo Tokyo Japan; 4 Department of Clinical Sciences Lund University Malmö Sweden; 5 Department of Diabetes and Metabolic Diseases Graduate School of Medicine The University of Tokyo Tokyo Japan; 6 School of Health Innovation Kanagawa University of Human Services Tonomachi Japan; 7 Clinical Biotechnology, Center for Disease Biology and Integrative Medicine Graduate School of Medicine The University of Tokyo Tokyo Japan

**Keywords:** computational epidemiology, COVID-19, SARS-CoV-2, agent-based modeling, public health, computational models, modeling, agent, spread, computation, epidemiology, policy

## Abstract

**Background:**

The spread of SARS-CoV-2, originating in Wuhan, China, was classified as a pandemic by the World Health Organization on March 11, 2020. The governments of affected countries have implemented various measures to limit the spread of the virus. The starting point of this paper is the different government approaches, in terms of promulgating new legislative regulations to limit the virus diffusion and to contain negative effects on the populations.

**Objective:**

This paper aims to study how the spread of SARS-CoV-2 is linked to government policies and to analyze how different policies have produced different results on public health.

**Methods:**

Considering the official data provided by 4 countries (Italy, Germany, Sweden, and Brazil) and from the measures implemented by each government, we built an agent-based model to study the effects that these measures will have over time on different variables such as the total number of COVID-19 cases, intensive care unit (ICU) bed occupancy rates, and recovery and case-fatality rates. The model we implemented provides the possibility of modifying some starting variables, and it was thus possible to study the effects that some policies (eg, keeping the national borders closed or increasing the ICU beds) would have had on the spread of the infection.

**Results:**

The 4 considered countries have adopted different containment measures for COVID-19, and the forecasts provided by the model for the considered variables have given different results. Italy and Germany seem to be able to limit the spread of the infection and any eventual second wave, while Sweden and Brazil do not seem to have the situation under control. This situation is also reflected in the forecasts of pressure on the National Health Services, which see Sweden and Brazil with a high occupancy rate of ICU beds in the coming months, with a consequent high number of deaths.

**Conclusions:**

In line with what we expected, the obtained results showed that the countries that have taken restrictive measures in terms of limiting the population mobility have managed more successfully than others to contain the spread of COVID-19. Moreover, the model demonstrated that herd immunity cannot be reached even in countries that have relied on a strategy without strict containment measures.

## Introduction

The spread of communicable diseases across a population is a spatial and temporal process, and the study of the transmission dynamics is becoming increasingly important for tackling the spread appropriately.

Agent-based models (ABMs) are a class of computational models based on computer simulations of actions and interactions of autonomous agents, aimed at evaluating how these actions affect the system as a whole. The agent-based approach emphasizes the importance of learning through the agent-environment interaction. This approach is part of a recent trend in the computational models of learning toward developing new ways of studying autonomous organisms in virtual or real environments.

ABMs have proven particularly useful for answering public health–related questions that are typically unanswerable with the traditional epidemiological toolkit [1]. The use of ABMs for studying phenomena related to public health is not recent and has been used to study the spread of alcohol consumption [2] and eating disorders [3].

Agent-based simulation modeling has been used primarily in epidemiological studies of infectious diseases, including the study of the reactions of the immune system during an infection [4], the spread of malaria following the movement of mosquitoes in a village in Niger [5], and following the trend of the influenza virus [6]. Additionally, ABMs have been used to study the trend of chronic diseases [7] and to analyze the public health impact of influenza vaccinations in the United States and their cost-effectiveness, simulating scenarios where different age groups of the population were vaccinated [8].

More recently, ABMs have been used in population-based studies of COVID-19, in particular to analyze the effects of population characteristics [9,10] and of public health measures on the spread of SARS-CoV-2 [11,12]. The importance of ABMs in the face of a global pandemic is their ability to reproduce situations, starting from real data, otherwise not reproducible in reality.

In this study, we propose an epidemiological ABM for analyzing the propagation of an infectious disease in a network of human contacts; in particular, our model studies the effects of political decisions on the spread of SARS-CoV-2. Other works have been done studying this aspect [13-15], but the approach was to simulate different pre-established situations (eg, implementing containment measures or performing many diagnostic tests), evaluating their impacts. A work similar to our study [16] starts from the same research questions and arrives at similar conclusions but uses a completely different methodology. Our study differs from the previous ones in that it analyzes the effects of the measures adopted by the governments in *real time* as they are implemented. An increasingly used ABM for modeling COVID-19 is Covasim [17]; although we propose a similar model that includes demographic information and nonpharmaceutical interventions, we considered a simplified network structure (specifically, a dynamic random network, where the edges are created and destroyed at each period *t*) with a focus on capturing only the stylized facts for an immediate evaluation of the effects of changes in model parameters and in policies.

The time stamp (in days) of the model accurately reflects the timing of the political decisions taken from the end of January to July 1, 2020, and the model studies the evolution of the virus from its appearance up to a year later. The parameters we defined were derived from government policies, from real data provided by the government bodies, and from medical knowledge about the virus up to July 1, 2020; beyond this date, the model makes predictions of how the virus would have spread if all the considered variables would have followed the same evolution (for example, maintaining the containment measures as of July 1 in the 4 countries). There was no knowledge at the time about virus variants nor data about the vaccination campaign, so these have not been included.

The hypothesis from which we start is that the spread of a virus depends, in addition to epidemiological factors and the nature of the virus itself, on individual behavior or, more precisely, on political decisions that induce appropriate behavioral criteria. Our goal is to show how, through targeted measures, the damage caused by the spread of a pandemic can be limited, both in terms of the case-fatality rate and pressure on hospitals.

## Methods

### Overview of the Model

We implemented the model using NetLogo (free and open-source software, released under a GNU General Public License; Rel. 6.1.0), a multi-agent programmable modeling environment (source code available on GitHub [18]). The simulation was performed using the data of 4 countries (Italy, Germany, Sweden, and Brazil) that have had different policy approaches for the containment of SARS-CoV-2.

Italy was chosen in our analysis as it was the first country (after China) to report an important diffusion of the virus in its territory and had to make new decisions and implement measures without having the possibility to compare their effectiveness with those of other similar countries. Germany followed the example of Italy but with a much higher execution speed, relying also on a greater number of intensive care unit (ICU) beds (the highest in Europe; Source: National Center for Biotechnology Information [19]). Sweden took a different approach from other European countries: it did not deny the presence and the potential consequences of the virus spread in its territory but decided not to impose any limitation to individual freedoms, essentially aiming at obtaining herd immunity. Like Sweden, Brazil did not adopt national measures to contain the spread of the virus, despite the high number of deaths that this has caused. A more detailed description of the differences and the reasons that led us to choose these 4 countries can be found in Multimedia Appendix 1.

The model studied, through the interactions between healthy individuals and infected individuals, how the virus spread over time and how the actions implemented by governments influenced its propagation. Starting from objective data provided by government bodies (Table 1), key variables related to the country, population, virus, and implemented policies were taken into consideration. We provide a complete list of the measured variables (Table 2; several indexes are the proportional transformation of the values obtained from Table 1, defined in the calibration phase of the model). We measured and demonstrated the results of how these variables evolved over time.

**Table 1:** Reference data used for constructing the model.

Demographics		Data (%)	Notes
**Italy**			
	Older than 65 years	22.60	Source: Eurostat [20]
	Beds for seriously ill patients	0.26	Source: OECD^a^ [21]
	Recovery rate	76.89	Source: World Health Organization [22]
	Case-fatality rate	14.55	Source: World Health Organization [22]
	Seriously ill	2.40	Source: Ministero della Salute [23]
	Hospitalization rate	25.40	Source: Ministero della Salute [23]
	Not seriously ill	74.60	Source: Ministero della Salute [23]
**Germany**			
	Older than 65 years	21.40	Source: Eurostat [20]
	Beds for seriously ill patients	0.60	Source: OECD [24]
	Recovery rate	91.15	Source: World Health Organization [25]
	Case-fatality rate	4.67	Source: World Health Organization [25]
	Seriously ill	1.48	Source: Worldometer [26]
	Hospitalization rate	6.20	Source: Worldometer [26]
	Not seriously ill	92.30	Source: Worldometer [26]
**Sweden**			
	Older than 65 years	19.80	Source: Eurostat [20]
	Beds for seriously ill patients	0.20	Source: OECD [27]
	Recovery rate	12.74	Source: Worldometer [26]
	Case-fatality rate	9.02	Source: World Health Organization [28]
	Seriously ill	2.55	Source: Worldometer [26]
	Hospitalization rate	25.68	Source: Worldometer [26]
	Not seriously ill	69.30	Source: Worldometer [26]
**Brazil**			
	Older than 65 years	8.60	Source: CIA^b^ [29]
	Beds for seriously ill patients	0.19	Source: AMIB^c^ [30]
	Recovery rate	49.81	Source: World Health Organization [31]
	Case-fatality rate	4.65	Source: World Health Organization [31]
	Seriously ill	2.00	Source: Worldometer [26]
	Hospitalization rate	8.00	Source: Worldometer [26]
	Not seriously ill	90.00	Source: Worldometer [26]

^a^OECD: Organisation for Economic Co-operation and Development.

^b^CIA: Central Intelligence Agency.

^c^AMIB: Associação Medicina Intensiva Brasileira.

**Table 2:** List of the model variables.

Variables	Italy	Germany	Sweden	Brazil
Total population (units)	1000	1000	1000	1000
Older than 65 years (units)	230	210	200	90
Initial infectious people (units)	6	6	6	6
Transmissibility rate (%)	0.30	0.30	0.30	0.30
Immunity duration: mild cases (days)	100	100	100	100
Immunity duration: severe cases	Lifetime	Lifetime	Lifetime	Lifetime
Initial productivity index	2.0	2.0	2.0	2.0
Noncontagion index	0.01	0.01	0.01	0.01
Virus recognition	After 100 cases	After 60 cases	After 100 cases	After 100 cases
Beds for seriously ill patients (units)	13	30	20	9
Recovery index	5.0	6.0	1.5	2.5
Case-fatality index	0.8	0.3	0.4	0.3
Seriously ill index	1.5	1.0	1.5	1.3
Not seriously ill index	5.0	5.0	4.7	6.0
Mask use (decrease in transmissibility; %)	14.3	14.3	0	7.15
Physical distancing (decrease in transmissibility; %)	10.2	10.2	10.2	5.1
Infected tourists (max number; units)	2	2	1	1

### Description of the Model

The model examined a sample of the population of each of the 4 countries, fixed at 1000 (*i* ∈ {1,2,...,1000}). It was similar to a small neighborhood of a city where the characteristics of the entire population are reproduced (the data we were interested in are reported in Tables 1 and 2). We assumed that an outbreak of COVID-19 has developed in this neighborhood. Naturally, government provisions were applied to this neighborhood as they were issued and with the same timing.

To better explain the logic behind our choices, we should imagine the considered space where the agents live as a laboratory where we applied the different policies, compliance to nonpharmaceutical measures, health structures, knowledge about the virus, etc. The *laboratory* space and the number of agents go beyond the geographical context, as they are meant to represent an exportable sample for each of the analyzed countries. Only the different measures and country specifications influence the results obtained from the simulations.

The time span of the simulation was 1 year, divided into 365 daily cycles. The first cycle coincided with the first infections in the given country.

The initialization of the model (at time *t*=0) requires the loading and setting of the required variables for the simulations and analyses. The variables derived from the national and governmental bodies, as well as institutional sources for each country, were automatically loaded following country selection. In this phase, the model also set the time stamps in which political decisions were made with respect to the containment measures for the spread of SARS-CoV-2. After the initial setting, the model was ready to simulate the evolution of COVID-19 for the selected country.

The following shows the (simplified) scheme followed by the model. The total 1000 agents move randomly within the model environment, simulating daily activities (eg, going to work or school); movement speed was set at a lower level (50%) for older adult agents (older than 65 years), as they perform fewer activities (the number of older adults was modeled according to the national statistics, see Table 2). Among the agents, some are infected (we denoted them as *I_i_*, and we fixed them at 6 at time *t*=0). Moving inside the environment, they come into contact with healthy agents. A healthy agent *H_i_* has a certain probability *P*(*I*) ∈ [0,1] to be infected, defined by the following equation:

*P*(*I*) = 1 – (1 – *TR*)*^n^*

where *TR* ∈ [0,1] is the transmissibility rate of the virus, and *n* is the number of infected neighbors; in our topology the number of infected agents present in a closed ball was determined by *B*_1_ = {*x* ∈ *R*^2^:‖x-y‖≤1}, with radius 1 and center *y* ∈ *R*^2^, where the healthy agent *H_i_* is located in time *t*. Notice that *P*(*I*) is monotonically increasing with respect to *TR* and *n*.

The propagation of the virus is not immediately recognized as such by the governments, and before this happens, the number of infected agents *I_i_* exceeds a certain threshold (see *virus recognition* in Table 2 for the country-specific threshold values).

After the virus is recognized, at each time *t*, we assume that each infected agent and those who have come into contact with them have a probability *P*(*test*)=0.5 to perform a virus recognition test [32]. Therefore, half of them do not perform the test and continue to move inside the model space becoming, if infected, a symptomatic infected agent or an asymptomatic infected one. Asymptomatic agents will perform the virus recognition test at the next period *t* + 1 only if they come into contact again with an infected agent, while symptomatic agents will have the same constant probability *P*(*test*) to be tested in each period.

Infected agents do not present symptoms immediately, but we considered that there is an incubation period that can vary according to age. For individuals younger than 65 years, we set the incubation period according to a normal distribution with mean 7 (SD 2; *I_Y_* ~ *N*(7,4)), while for those who are older than 65 years, the incubation period is defined according to a normal distribution with mean 3 (SD 1; *I_O_* ~ *N*(3,1)).

The viral load, and therefore the ability to infect other agents, has not been set the same for all of the *I_i_* agents. For those who are in the incubation phase, the viral load is lower, and it increases period by period as the development of the infection approaches; for asymptomatic agents, it is lower than for agents with mild symptoms, who in turn, will have it lower than seriously ill agents (that will need to be hospitalized).

Infected agents *I_i_* can therefore be of four types: in incubation, asymptomatic, mildly ill, and seriously ill (see Tables 1 and 2). The mildly ill, when found, are isolated at home; in our model, this translates to their mobility being set to 0 (but they can still spread the virus). The seriously ill, when found, will be hospitalized, and their mobility will also be set to 0. Furthermore, the latter are to be considered in an isolated space, so we also considered that they will not spread the virus anymore.

If ICU beds (see *beds for seriously ill patients* in Table 2) are saturated, seriously ill patients will be placed in home isolation (with mobility at 0), but their probability of recovery (see Tables 1 and 2) decreases.

Seriously ill patients can die with a probability equal to that listed in Table 1 (*case-fatality rate*) and Table 2 (*case-fatality index*). For older adults, this probability is higher (we consider their greater fragility and the possible presence of other existing pathologies). Dead agents are denoted with *D_i_*; we set both their mobility and their transmissibility rate to 0.

Ill patients can recover with a probability equal to that listed in Table 1 (*recovery rate*) and Table 2 (*recovery index*).

Recovered agents develop antibodies to the virus (ie, they become immune). We denoted the immune agents with *IM_i_*. For those who were seriously ill, we considered that their antibodies lasted for the whole simulation, while those who were mildly ill will develop an immunity that lasts only for 100 days [33] (see Table 2). Recent studies [34] confirm that there is a difference in the duration of immunity, which depends on the severity of the development of the disease.

We also considered noncontagious asymptomatic cases (ie, there is a small proportion of healthy individuals who, following infection, immediately develop antibodies without showing symptoms and never become carriers of the virus). They transition from *H_i_* in *t* to *IM_i_* in *t* +1 and are not counted as *I_i_*. Notice that in each *t*, the sum of all the agents (healthy, infected, immune, and dead) is equal to 1000.

The industrial productivity (economic index) is proportional to the mobility of the agents. By setting the prepandemic level to 0, the reduced mobility of the agents will lead to a decrease in productivity.

**Figure 1:**
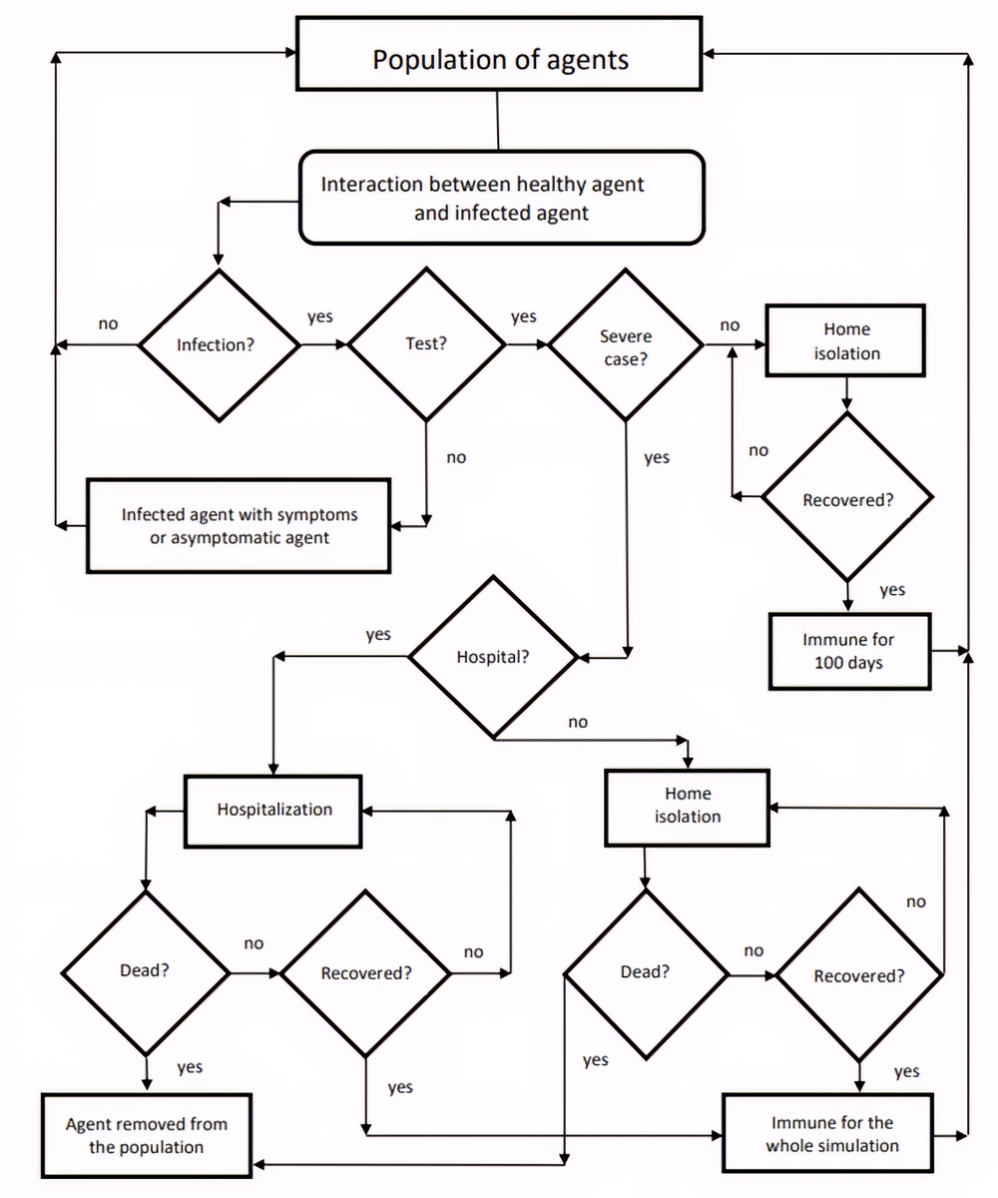
Simplified flowchart of interaction mechanisms in the model.

Figure 1 shows a simplified flowchart of the mechanisms previously described.

Political decisions were then applied to this scheme, according to the times and the ways they have been implemented by the governments of the analyzed countries. Thus, for example, the decision of closing schools will lead to a reduction in the initial mobility of the agents; a lockdown of nonessential activities will further reduce it (with negative repercussions on industrial productivity, but a positive result in terms of limiting the spread of the contagion). The adoption of precautions or medical aids was translated into the model as a decrease in the transmissibility rate (see Table 2). We analyzed only a small sample of the population; therefore, in the rest of this study, the political decision of closing the national borders was translated into a further limitation on the mobility of agents, while their reopening was simulated as a partial restoration of the original mobility and the introduction of new infected agents (see *infected tourists* in Table 2).

A more detailed explanation of the parameters and the variables we used can be found in Multimedia Appendix 1.

## Results

It should be remembered that the reported results were obtained considering the government measures in force until July 1, 2020; from this date onward, the forecasts are based on the last known measures being kept in place. In the summer of 2020, individual behavior and governments’ attitudes were not so strict: therefore, despite the model correctly forecasting a second wave, such forecasts were underestimated.

### Italy

The simulation recorded 309 cases of COVID-19 with 243 recoveries and 48 deaths. The case-fatality rate was 15% (48/309), with an older adult (older than 65 years) case-fatality rate of 65% (31/48).

#### Total Number of COVID-19 Cases, Hospitalizations, Isolations, and Asymptomatic Infections

**Figure 2:**
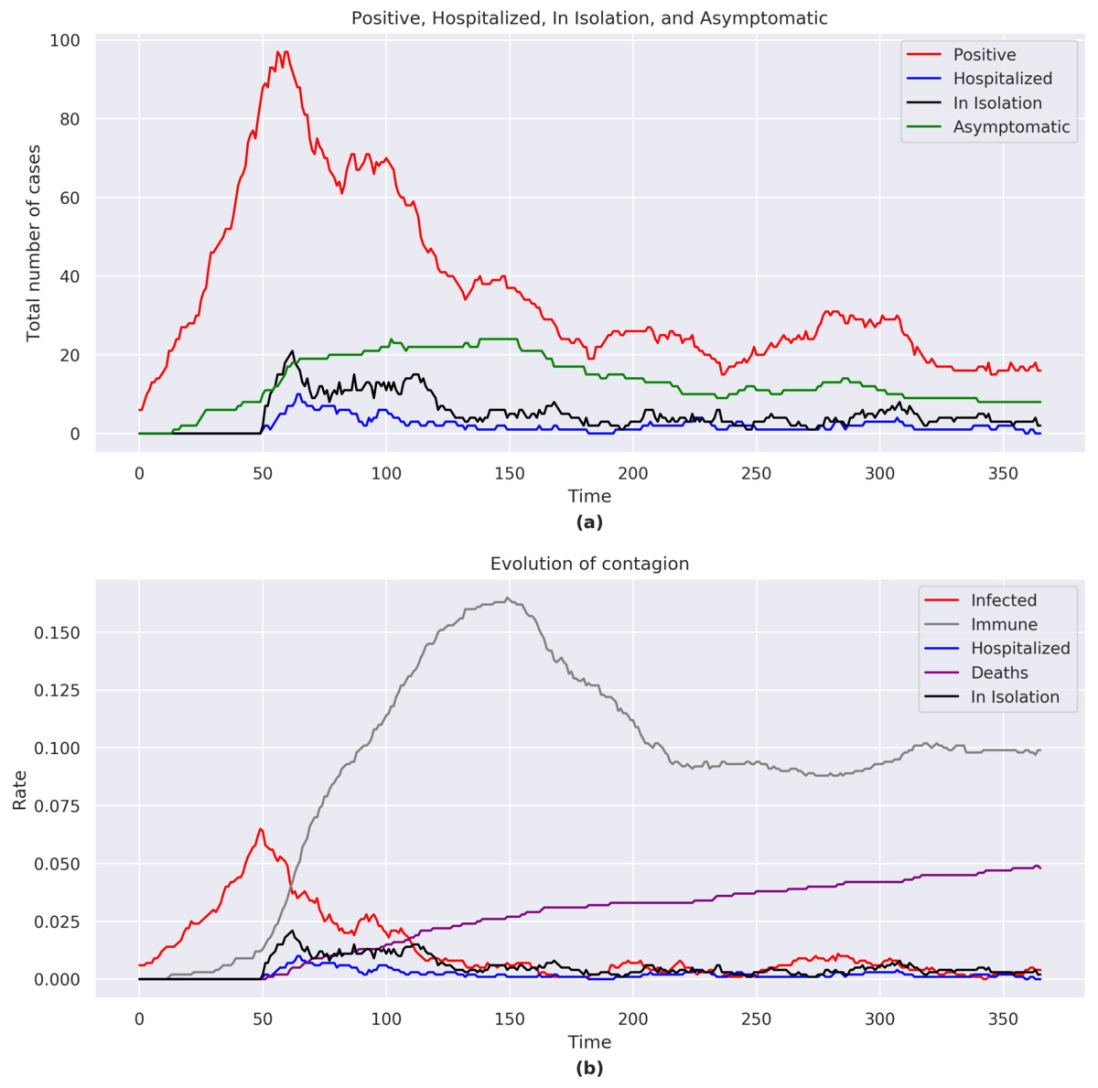
(a) Positive, hospitalized, in isolation, and asymptomatic figures for Italy. (b) Evolution of the contagion for Italy. The graphs consider the sum of the agents belonging to each category shown in the legend for each day of the simulation.

At the end of the simulation, the total number of positives was 17, including 8 asymptomatic, 2 in home isolation, and 0 hospitalizations. The number of COVID-19 cases rose exponentially with a peak at *t*=61 (Figure 2a). In the second half of the simulation (*t*=279), the number of COVID-19 cases rose but never reached the height of the initial peak.

The total number of hospitalizations and home isolations reached a peak at *t*=64.

#### Immunity and Case Fatality

The proportion of individuals who acquired immunity reached a peak of 16.5% (165/1000) at *t*=150, albeit with an immunity of 10% (99/1000) at the end of the simulation (Figure 2b). The case-fatality rates increased throughout the simulation despite a decreasing number of cases. At the end of the simulation, the case-fatality rate was 4.8% (48/1000).

#### R_0_ and R_E_

**Figure 3:**
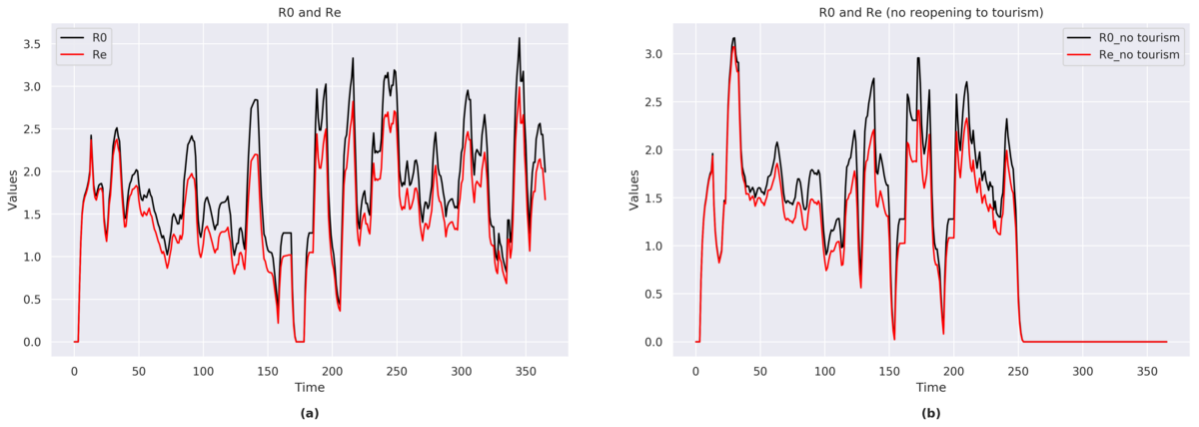
(a) R_0_ and R_e_ indexes for Italy (with national borders reopening). (b) R_0_ and R_e_ indexes for Italy (with no national borders reopening).

The trends of R_0_ (range 0-3.5) and R_E_ (range 0-3.0) exhibited strong fluctuations during the time span of the simulation (Figure 3a).

A sensitivity analysis conducted in a simulation that assumed that national borders remained closed (Figure 3b) showed that no new cases occurred once the *no contagion* value was reached, and the borders remained closed.

#### Herd Immunity, ICU Beds, and Productivity

The model showed that immunity was reached in approximately 11% (116/ 1000) of the population.

The simulation indicated that the ICU beds were never saturated; although at *t*=66, the occupancy rate reaches 77% (10/13).

The model showed a sharp drop in productivity following the implementation of containment measures, and the loss in productivity at its maximum reached –18.7% (compared to the prepandemic value of 0).

### Germany

The simulation recorded 270 cases of COVID-19 with 233 recoveries and 18 deaths. The case-fatality rate was 6.7% (18/270), with an older adult case-fatality rate of 61% (11/18).

#### Total Number of COVID-19 Cases, Hospitalizations, Home Isolations, and Asymptomatic Infections

**Figure 4:**
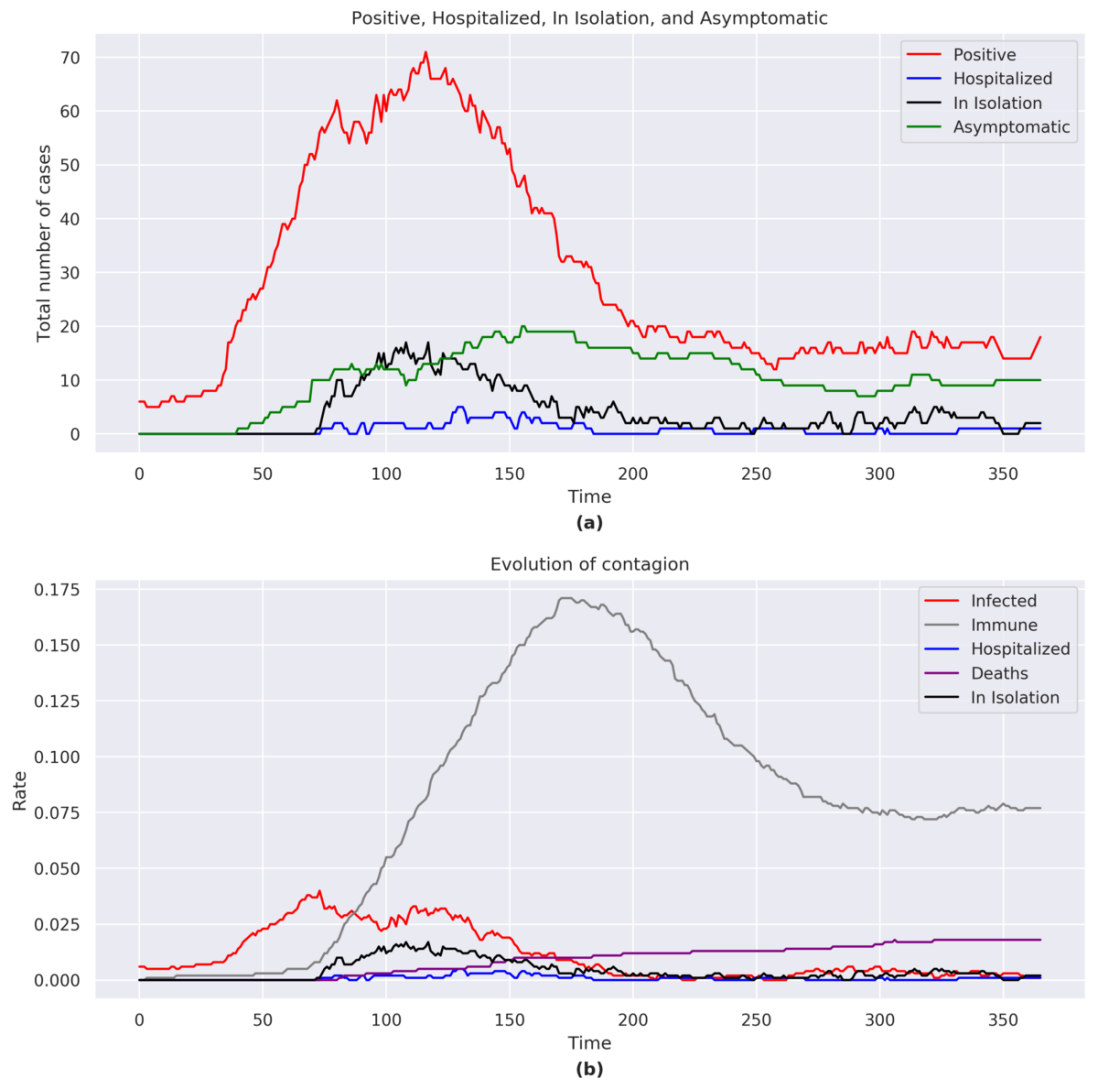
(a) Positive, hospitalized, in isolation, and asymptomatic figures for Germany. (b) Evolution of the contagion for Germany. The graphs consider the sum of the agents belonging to each category shown in the legend for each day of the simulation.

At the end of the simulation, the total number of positive cases was 19, including 10 asymptomatic, 2 in home isolation, and 1 hospitalized. The number of COVID-19 cases rose rapidly with a peak at *t*=117 (Figure 4a). After the peak and following the adopted containment measures, the number of COVID-19 cases gradually decreased.

The total number of hospitalizations and home isolations reached a peak around *t*=110.

#### Immunity and Case Fatality

The proportion of individuals who acquired immunity reached a peak of 17.1% (171/ 1000) at *t*=175, albeit with an immunity below 8% (77/1000) at the end of the simulation (Figure 4b). The case-fatality rate increased throughout the simulation. In the last part of the simulation, despite new cases of COVID-19, the case-fatality rate did not increase. At the end of the simulation, the case-fatality rate was 1.8% (18/1000).

#### R_0_ and R_E_

The trends of R_0_ (range 0-3.8) and R_E_ (range 0-3.0) exhibited strong fluctuations during the time span of the simulation.

The sensitivity analysis showed that no new cases occurred once the *no contagion* value was reached (same as the analysis for Italy; Figure 3b), and the borders remained closed. Conversely, in a situation with open national borders, COVID-19 was not completely eradicated despite the implemented measures.

#### Herd Immunity, ICU Beds, and Productivity

The model showed that immunity was reached in approximately 10% (96/1000) of the population.

The simulation indicated that the ICU beds were far from being saturated, with the highest rate being 17% (5/30) at *t*=131.

The model showed a sharp drop in productivity following the implementation of containment measures, with a maximum loss of productivity of –18.2% (compared to the prepandemic value of 0).

### Sweden

The simulation recorded 765 cases of COVID-19 with 533 recoveries and 141 deaths. The case-fatality rate was 18.4% (141/765), with an older adult case-fatality rate of 43% (61/141).

#### Total Number of COVID-19 Cases, Hospitalizations, Home Isolations, and Asymptomatic Infections

At the end of the simulation, the total number of positive cases was 91, including 38 asymptomatic, 29 in home isolation, and 9 hospitalized.

**Figure 5:**
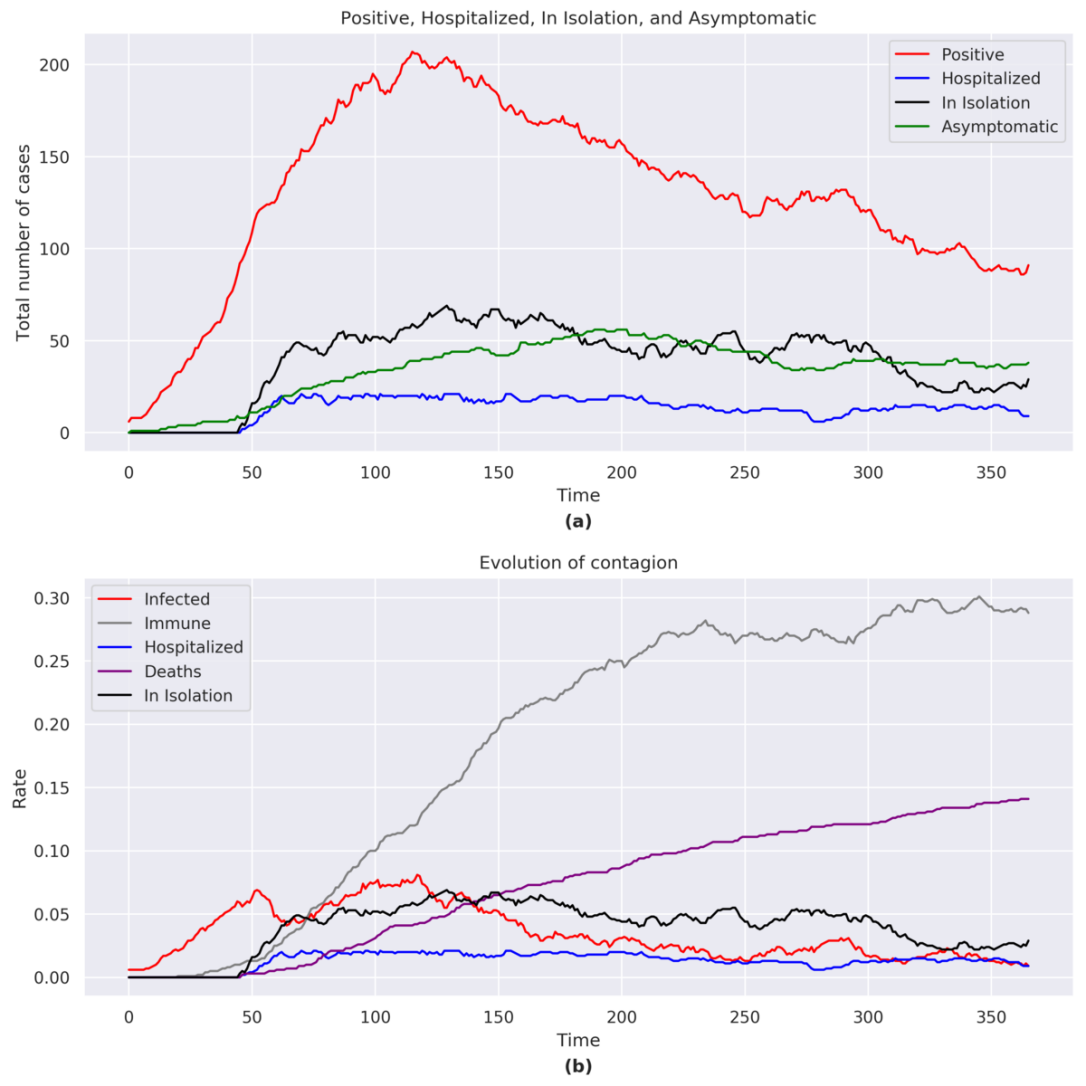
(a) Positive, hospitalized, in isolation, and asymptomatic figures for Sweden. (b) Evolution of the contagion for Sweden. The graphs consider the sum of the agents belonging to each category shown in the legend for each day of the simulation.

The number of COVID-19 cases reached a peak at *t*=116 (Figure 5a) with the number of cases decreasing slowly, remaining at high values until the end of the simulation. Despite a descending trend in the second half of the simulation, there were situations where the number of cases increased again. Given the high number of positive cases, the infection was not considered under control.

The total number of hospitalizations and home isolations reached a peak around *t*=129.

A sensitivity analysis that did not place a limit on the number of ICU beds showed that in the second part of the simulation there was a sharper decrease, with an overall lower number of COVID-19 cases.

#### Immunity and Case Fatality

Immunity was reached in 29% (290/1000) of the population, with a COVID-19 mortality rate of 14% (141/1000) at the end of the simulation (Figure 5b). The recovery rate was 60% (443/733; we did not count the number of positive agents at the end of the simulation).

The sensitivity analysis showed that an increase in the number of ICU beds would lead to a decrease in the total number of positive cases and would result in an increased recovery rate (447/588, 76%; we did not count the number of positive agents at the end of the simulation) and a decreased case-fatality rate (80/693, 11.5%).

#### R_0_, R_E_, and Herd Immunity

**Figure 6:**
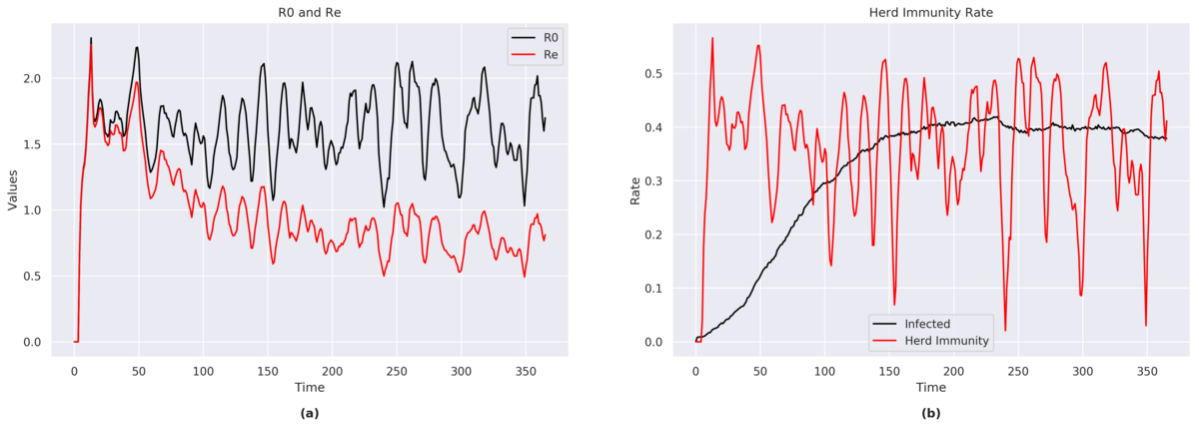
(a) R_0_ and R_e_ indexes for Sweden. (b) Herd immunity rate for Sweden.

The trends of R_0_ (range 1.1-2.3) and R_E_ (range 0.5-2.2) presented less fluctuations during the time span of this simulation (Figure 6a).

The model showed that immunity was reached in approximately 39% (388/1000; we counted the immune plus the currently positive cases) of the population (Figure 6b).

#### ICU Beds

**Figure 7:**
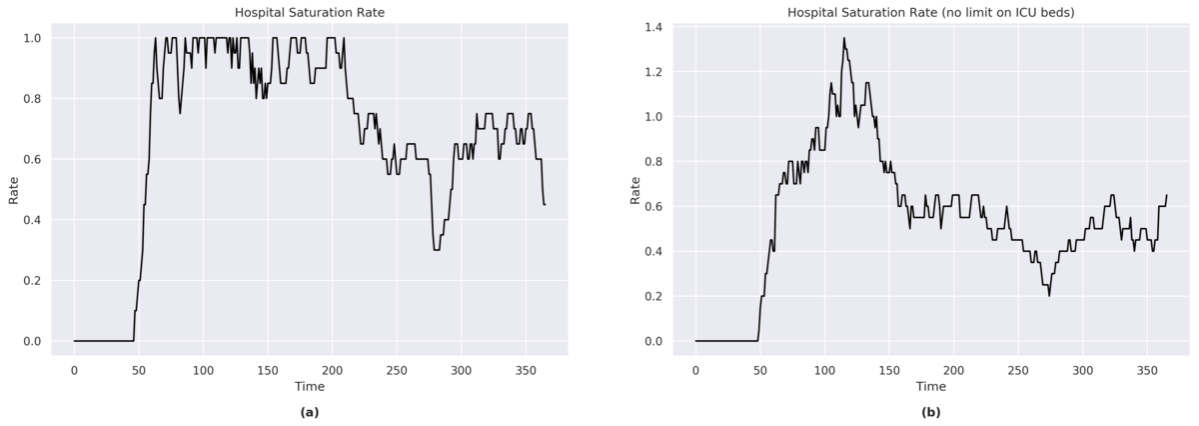
(a) Hospital saturation rate for Sweden. (b) Hospital saturation rate for Sweden (with no limit on the number of ICU beds). ICU: intensive care unit.

In the first part of the simulation, full saturation of ICU beds was reached on a number of occasions (Figure 7a). In the second part of the simulation, the bed saturation rate remained above 50%.

The sensitivity analysis showed that approximately an additional 40% (an increase of 8 out of the current 20) of the available ICU beds would have been necessary to cope with the peak of a maximum emergency (at *t*=116; Figure 7b). An additional 10% of available beds would have met the needs of the population throughout most of the simulation period.

#### Productivity

The loss in productivity at its maximum reached –18.8% (compared to the prepandemic value of 0). The government measures have generated a decrease in productivity, and as these measures were still in place as of July 1, 2020, we could not see a rise due to the restoration of normality. The loss of productivity was mainly affected by the high number of infected individuals in hospital and in home isolation, and the high case-fatality rate.

In the sensitivity analysis without a limit on ICU beds, the productivity was higher than in the main analysis.

### Brazil

The simulation recorded 883 cases of COVID-19 with 658 recoveries and 90 deaths. The case-fatality rate was just over 10% (90/883), with an older adult case-fatality rate of 34% (31/90) out of total deaths.

#### Total Number of COVID-19 Cases, Hospitalizations, Home Isolations, and Asymptomatic Infections

At the end of the simulation, the total number of positive cases was 137, including 63 asymptomatic, 47 in home isolation, and 3 hospitalized.

**Figure 8:**
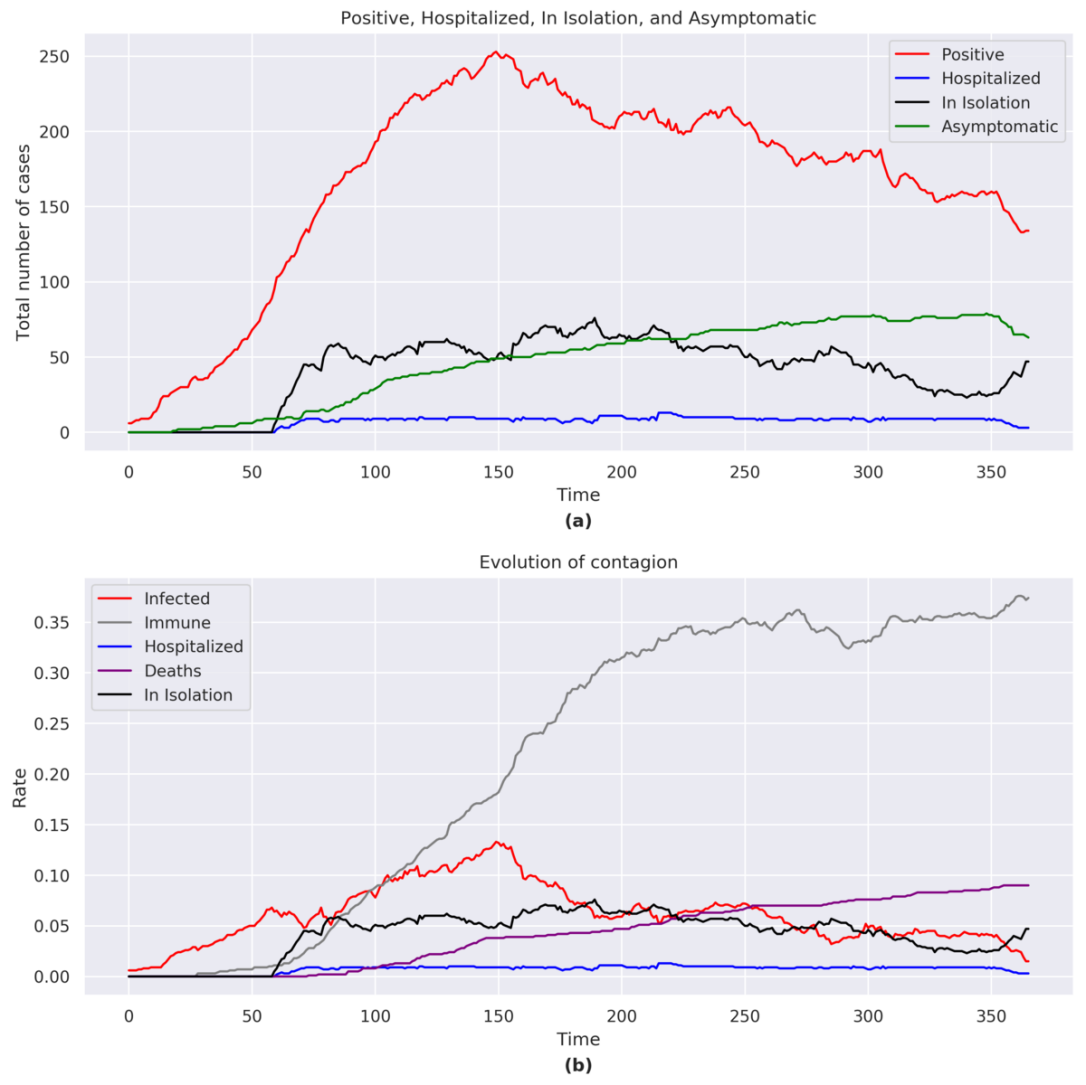
(a) Positive, hospitalized, in isolation, and asymptomatic figures for Brazil. (b) Evolution of the contagion for Brazil. The graphs consider the sum of the agents belonging to each category shown in the legend for each day of the simulation.

The number of COVID-19 cases gradually rose throughout most of the simulation period without reaching a clear peak. The final part of the simulation showed that there was a decrease in the number of cases due to the large number of immune individuals. Given the high number of people still positive at the end of the simulation, the infection was not to be considered under control (Figure 8a).

The total number of hospitalizations and home isolations reached a peak around *t*=190.

A sensitivity analysis that did not place a limit on the number of ICU beds resulted in a peak of the number of COVID-19 cases at *t*=75, followed by a gradual decrease in the number of cases.

#### Immunity and Case Fatality

The proportion of immune individuals reached 37% (374/1000) of the population (Figure 8b). The proportion of recovered cases reached 74% (658/883).

The sensibility analysis showed that an increase in the number of ICU beds would lead to a decrease in the number of cases and would result in a recovery rate of 84% (415/492) and a case-fatality rate of 7% (34/492).

#### R_0_ and R_E_, Herd Immunity, ICU Beds, and Productivity

The trends of R_0_ (range 0.5-4.1) and R_E_ (range 0.2-2.5) exhibited contained fluctuations during the time span of this simulation but always remained higher than 1. 

The model showed that immunity was reached in approximately 51% (511/1000) of the population, while a figure of 75% was required to obtain herd immunity for the entire population.

**Figure 9:**
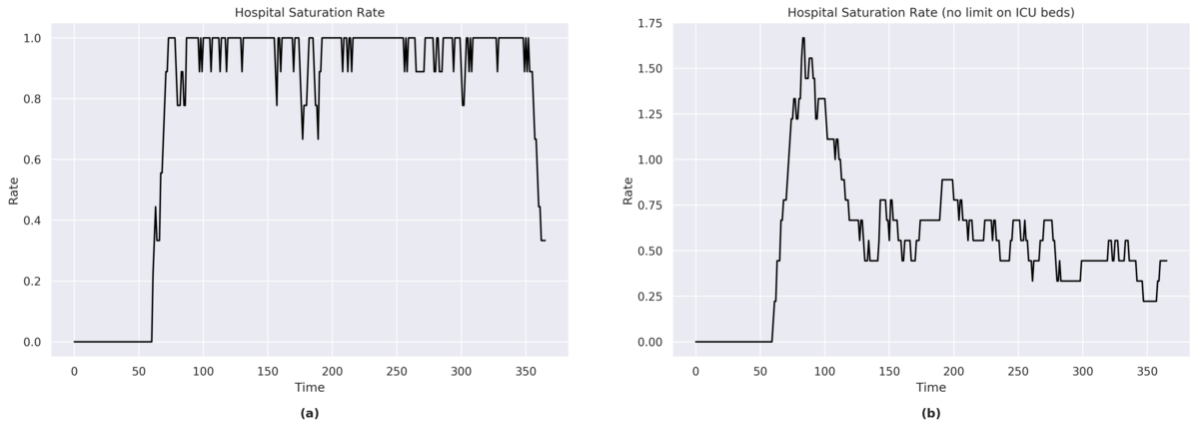
(a) Hospital saturation rate for Brazil. (b) Hospital saturation rate for Brazil (with no limit on the number of ICU beds). ICU: intensive care unit.

The simulation model showed that the number of ICU beds were insufficient with respect to the needs resulting from the spread of the COVID-19 pandemic (Figure 9a).

The sensitivity analysis, which removed the limit on the number of ICU beds, showed that, approximately, an additional 75% (an increase of 7 out of the current 9) of the number of available ICU beds would have been necessary to cope with the peak of maximum emergency (at *t*=84; Figure 9b).

The loss in productivity at most reached –12.4% (compared to the prepandemic value of 0).

## Discussion

### Objective

The objective of this study was to present a model that simulates the propagation of the COVID-19 pandemic based on real-world containment measures, as they were implemented by the governments of 4 countries: Italy, Germany, Sweden, and Brazil. The model thus allows for a prediction on the evolution of COVID-19 by reporting forecasts on key indexes such as the case-fatality rate, the recovery rate, herd immunity, ICU bed occupancy rates, home isolation rates, and the countries’ productivity rates. The proposed model is highly flexible and allows for the addition or removal of parameters such as requirements and policies. Moreover, the model consequently studies how the contagion evolves over time. This offers the possibility to run additional simulations that predict the course of the pandemic under alternative policies by each government.

Previous models of SARS-CoV-2 have assessed the impact of the use of personal protection and early diagnosis [11], studied the impact of face masks on the spread of the virus [12], and analyzed the impact of the virus according to age [9,10], family situation, and the presence of comorbidities [10]. Meanwhile, other ABMs have considered the impact of home isolation on the saturation of ICU beds [35], assessed infection and fatality rates assuming a 20-fold underreported number of cases [36], or hypothesized the economic effects in Japan of a Tokyo lockdown [37]. Our model thus differs from previous models, as it focuses on the effects of contagion and on its evolution over time, considering both the real data made available by government bodies and the policy measures implemented to stop or limit the propagation of SARS-CoV-2.

The model outputs shown in this paper are the results of several simulations for each country. Due to the nature of ABMs, the quantitative results will differ with each simulation. Any conditions that occur within the model will vary over time while maintaining parameter values and keeping initial variables constant. Although each simulation will not yield identical quantitative results for each country, the qualitative behavior always follows the same trend. Consequently, we have been able to draw some considerations about the analyzed parameters. These are presented on a country by country basis.

### Italy

The simulations for Italy show a low total number of COVID-19 cases compared to the simulations for Sweden and Brazil, indicating a success of the adopted containment measures. Similarly, the numbers of hospitalized individuals and those in home isolation seemed to remain under control. Overall, this resulted in a large fluctuation of R_0_ and R_E_, where a small increase in the number of infections lead to a large growth in the indexes’ values. Additionally, the simulations for Italy indicated a slow reduction in the number of asymptomatic individuals, which highlights an increased possibility of new infections that in turn could be extended to a recommendation not to loosen the implemented containment measures as of July 1, 2020. This is further supported by the trends in immunity, where the proportion of immune individuals were comparatively low. Bearing in mind that our model was implemented at the end of June 2020 and that it used statistical data available at that time, it was able to correctly predict that, with the reopening of the national borders and the free movement of people, there would be a new increase in the number of positive cases (thereby partly invalidating national containment efforts). This was indeed confirmed in our sensitivity analysis where the national borders remain closed. Such a situation is not replicable in reality, but it leads to no new cases.

Despite its relatively low number of cases, Italy recorded the second highest case-fatality rate (48/309, 15%; Germany: 18/270, 6.7%; Sweden: 141/765, 18.4%; Brazil: 90/883, 10%). Italy’s proportion of older adults ranks among the highest in the world (Source: Istituto Nazionale di Statistica [38]), which could serve to partially explain the exceedingly high case-fatality rate. Indeed, the simulation indicates that COVID-19 affects older adults predominantly, where the case-fatality rate reached 65% (31/48) of total deaths.

Herd immunity in Italy would be obtained in a situation where 70% of the population are immune to SARS-CoV-2. The results of the model, however, indicated that only 11% of the Italian population reached immunity, a number that considers both immune individuals and active cases. This low proportion of immune individuals is expected given the policy decisions aimed at limiting the spread of the virus.

### Germany

For Germany, our model was able to make a complete analysis of the contagion peak and its gradual descent, as well as predicted possible developments in the coming months.

The simulations for Germany showed a situation with a comparatively low number of infected individuals and strong fluctuations in R_0_ and R_E_ indexes. The low infection rates resulted in a very low case-fatality rate; however, it also resulted in a low proportion of immune individuals. Like the simulations for Italy, the low number of positive cases and the low proportion of immune individuals was a consequence of the policy implementations aimed at containing the spread of SARS-CoV-2. Overall, the results at the end of the simulation for Germany are not too different from the results obtained for Italy, a situation under control with regard to hospitalizations and home isolation cases. Moreover, as for Italy, asymptomatic cases were still recorded (1%), which indicates a situation where SARS-CoV-2 is still present in the population, with the risk of continued virus spread if the containment measures were to be loosened. The model, starting from the data at the end of June 2020, correctly predicted that this percentage of asymptomatic people would have led to the formation of new outbreaks and a relatively new spread of the virus.

The simulations for Germany showed two notable differences compared to Italy. First, the proportion of recoveries was higher in Germany (233/251, 93% vs 243/292, 83%). Second, although the German simulations showed an older adult case-fatality rate of 61% (11/18), the overall case-fatality rate was only 6.7% (18/270). Germany’s markedly lower overall case-fatality rate as compared to Italy could be the result of the prompt diagnosis and case management due to the widespread controls carried out by the public health authorities. The same containment measures, however, also result in the low proportion of immune individuals (96/1000, 10%), which are far from the proportion necessary to reach herd immunity as indicated by the model (73%). Consequently, and similar to Italy, the model predicted that Germany would have had a high risk of possible *second waves*, as it indeed happened with the reopening of the national borders.

Compared to Italy, Germany also fared better with regard to ICU bed occupancy rates; the simulations indicated that even in the most acute phase of the pandemic, bed occupancy rates never exceeded 20% (6/30) of total capacity. It should be kept in mind that Germany has by far the highest number of ICU beds in the 4 countries considered in our analysis [18].

Finally, the impact of the pandemic on the German economy is evident, as the containment measures had a strong impact on the productivity, which at one point reached –18.2%. However, contrary to the situation for Italy, the forecasts of major economic institutes such as the OECD and the World Bank considered the German recovery period to reach the precrisis values quicker than Italy.

### Sweden

The simulations for Sweden demonstrate a situation that is not under control a year after the first recorded case and are thus in stark contrast to those obtained for Italy and Germany. These discrepancies are most likely due to the comparatively limited containment measures initiated by the Swedish Public Health Authority. First, both R_0_ and R_E_ remain at higher values through the simulations, with an R_0_ that never goes below 1. Second, at the end of the simulation, the total number of COVID-19 cases was much larger than in Italy and Germany; the high number of hospitalized and asymptomatic cases being of particular concern. Third, the proportion of recovered cases (443/733, 60%) was lower than the corresponding proportion in any of the other countries. Fourth, the case-fatality rate (141/765, 18.4%) was higher than the rates obtained for Germany, Italy, and Brazil.

A major difference of Sweden from Italy and Germany was the low number of available ICU beds. Despite its high focus on welfare, Sweden has a low number of ICU beds per capita. Although Sweden managed to double the number of ICU beds at the start of the pandemic (Source: Folkhälsomyndigheten [39]), the pressure on hospitals remains critical throughout the simulations. Indeed, as the sensitivity analyses showed, Sweden would have required an additional 40% of its ICU capacity at the peak of the pandemic. Moreover, the sensitivity analysis also showed that the case-fatality rate decreased from 18.4% to 11.5% with a higher number of ICU beds. It is therefore fair to conclude that an increase in the ICU capacity would have the potential to save many lives.

Despite the adverse outcomes, Sweden does not reach the threshold of herd immunity as determined by the simulations. The herd immunity threshold (57%) was derived based on specific considerations in the model (ie, lifelong immunity for those with serious COVID-19 and temporary immunity to those with milder forms of the disease). To the best of our knowledge, there is no clear data on immunity. Indeed, if the parameters in the model are accurate, herd immunity for COVID-19 would be difficult to reach. It is therefore possible to conclude, based on the simulations, that implementing containment measures and recommending the use of face masks have positive effects in limiting the spread and the consequences of COVID-19, even a year after the first recorded case.

The simulated productivity drop for Sweden would, unlike the situations in Italy and Germany, not be influenced by the country’s containment measures but rather be a consequence of its large number of COVID-19 cases.

### Brazil

As expected, our model foresees that Brazil has the highest number of COVID-19 cases among the 4 analyzed countries. This is most likely due to Brazil’s implemented policy decisions, which are more in line with those of Sweden than Italy and Germany. Consequently, Brazil and Sweden share many similarities in the analyses. First, the total case numbers in Brazil resemble those of Sweden and are assumed to be a result of the less restrictive containments measures. Moreover, R_0_ and R_E_ did not exhibit strong fluctuations, with R_0_ remaining above 1 for the duration of the simulation. Despite a situation that could be considered out of control, Brazil displayed a notably lower case-fatality rate than Sweden (10.2% vs 18.4%). This is most likely due to the low proportion of older adults (8.6% vs 19.8% in Sweden). Another noteworthy difference between Brazil and Sweden was the encouraging recovery rate of 74% (658/883), again most likely due to the two countries’ demographic differences in age.

The proportion of immune individuals in Brazil reached 37% (374/1000) of the population, the highest proportion of all the analyzed countries. Despite this high immunization rate, the model foresees that herd immunity will not be reached due to a calculated threshold of 75%. Indeed, the total proportion of immunized and positive cases at the end of the simulation reached 51% (511/1000).

The severity of the situation in Brazil was further highlighted by the sensitivity analyses, identifying a required 75% increase in the number of ICU beds for Brazil to cope with its situation. According to the models, such an increase in capacity would notably reduce not only the number of recorded COVID-19 cases but also the case-fatality rate (from 10.2% to 7%). It is, however, questionable whether an ICU capacity increase of such magnitude is feasible to implement, as Brazil over the past years has progressively decreased the availability of ICU beds (Source: Central Intelligence Agency [29]).

Brazil has adopted few measures concerning the closure of commercial activities (Source: Conselho Nacional de Secretários de Saúde [40]). This led to a lower drop in productivity (in absolute terms) than in European countries, with the difference that the contagion curve in Brazil lowers slowly; the model predicted that the negative effects of the pandemic will last for a long time so that it seems likely that other countries (that implemented stronger containment measures) will be able to reopen all their activities sooner. The productivity trend reflects this, as there is no rise toward pre–COVID-19 values.

The effects of reopening national borders cannot be assessed for Brazil, which unlike Germany and Italy, has never implemented closure of national borders as a measure to contain the spread of COVID-19.

### Principal Results

By considering 4 countries with different policy approaches in the prevention and containment of the spread of COVID-19, our simulation model is able to highlight the consequences of policy decisions on a number of measures. The results obtained from our models showed the importance of prevention through widespread testing over large areas of territory (Germany) and of lockdown measures for the reduction of virus transmissibility (Italy and Germany). On the other hand, the countries that have not adopted these measures (Sweden and Brazil) are facing a situation that is not under control. From our results, we also highlight how important the mandatory use of face masks and the imposition of physical distancing are in reducing the number of COVID-19 cases. Our study also stresses how important it is to have an adequate number of ICU beds to deal with emergencies. This is evident particularly in the simulations for Sweden and Brazil, where the sensitivity analyses demonstrated an improvement in both recovery rates and case-fatality rates. Finally, the simulations showed that the reopening of national borders will not allow individual countries to maintain a monotonic decreasing curve of infections; indeed, only the simulations with the national borders being kept closed led to a complete stop of the spread of COVID-19.

In the context of an increasing number of positive COVID-19 cases, the main priority is the successful containment of the spread of SARS-CoV-2. However, prolonged lockdown measures have devastating effects on the economy of a country. The results of our model point toward a situation where countries that implemented mild policies against the virus at the start of the pandemic may inevitably need to strengthen them in the near future. Consequently, we suggest that the best course of action is to plan and implement aggressive political actions, both in the contagion containment phase (eg, limitations on the personal mobility and closure of nonessential activities) and in the economic recovery phase (eg, strong tax breaks for businesses and robust actions to stimulate consumption, as also indicated by the European Central Bank, even if doing this will result in a large budget deficit), with a long-term perspective from the beginning. According to the simulations, such actions may allow nations to overcome the economic impact of the pandemic sooner. This is important given that the data provided by the international economic organizations (International Monetary Fund, Organisation for Economic Co-operation and Development [OECD], World Bank, and others) leave no room for optimism [41-43].

### Strengths and Limitations

There are a number of limitations that need to be mentioned. The main point concerns the input data for the model. We have retrieved the values from the most reliable sites among those that provide daily information about the spread of the virus, but this information is constantly evolving. Consequently, to keep the model updated, it is necessary to set up the most recent information. In this paper, the model *photographs* the situation at the end of June 2020, and it provides a forecast based on those data. Another limitation is that we considered only a small sample (which can be thought of as an infection outbreak). Even if this sample has the same national characteristics, the obtained results may not perfectly be the same when translated on a larger scale; that said, what we have obtained remains valid when studying a representative outbreak.

The economic results obtained from the model measured only the impact resulting from political decisions to contain the spread of COVID-19. The economic ramifications that will occur after a complete reopening of borders, such as a decrease in consumption and tourism, an increase in unemployment, and the shutdown of various economic activities, have not been taken into consideration.

The simulations also have a number of strengths. They take into consideration the age distribution of the respective countries. This is crucial given the impact of COVID-19 on the older adult population. The data in all the simulations is based on official statistics, as they are obtained through the national statistical databases of each country. This is a major strength for Sweden, Germany, and Italy, but a limitation for the analyses relating to Brazil. Moreover, the model can be extended to include additional new and relevant variables as they become available or are deemed necessary by researchers and policy makers.

### Future Considerations

Further development of the model could allow for comparisons of the outcomes of a number of different policy proposals (eg, obligatory vs voluntary use of face masks, whether or not to increase the number of ICU beds, or whether or not to implement lockdown measures). The model could therefore be used to evaluate the needs and requirements for the considered territory, and the policies with the greatest impact over time. We plan to better explore these points in future research.

Additionally, with regard to the economic consequences of the pandemic, further considerations should be made for data concerning productivity and the economy in general. At the time of writing, the return to a situation similar to the one before the pandemic seems likely to occur only after the vaccination campaign ends, covering at least 75% of the population [44].

### Conclusions

This study used real-world data to analyze how different political decisions aiming to deal with the spread of SARS-CoV-2 influence the extent of COVID-19. The results of the simulations lead to three main conclusions. First, strict containment measures, including the mandated use of face masks and the implementation of social distance, lead to a reduction in the number of COVID-19 cases. Second, the number of ICU beds are an important measure to reduce case-fatality rates. Third, herd immunity cannot be reached, and any national strategy aiming to reach herd immunity by loosening containment measures should be avoided.

## Appendix

Multimedia Appendix 1 Supplementary material.

## Abbreviations

ABM: agent-based model
ICU: intensive care unit
