# Supplementary material for: An Agent-Based Model of the Local Spread of SARS-CoV-2: Modeling Study
Source: JMIR Med Inform. 2021 Apr 6;9(4):e24192. doi: 10.2196/24192 (PMC8025915; doi:10.2196/24192)
Supplement: Multimedia Appendix 1 [file medinform_v9i4e24192_app1.docx]

## **Multimedia Appendix 1.** Supplementary material.

## Methods

## Selected countries

### Italy

Italy was the first European nation to face the emergency of the COVID-19 spread. After an initial phase of uncertainty about the measures to be taken, it adopted very strong measures to contain the spread, starting from closing the schools up to the closure of all non-essential activities and the restriction of circulation between the municipalities; it has also been imposed to respect physical distance and the use of face masks. Thanks to the positive results obtained, gradual reopening proceeded until a complete recovery of the activities, still maintaining the obligation of physical distancing, and wearing face masks in closed spaces.

### Germany

Germany was one of the first European nations to face the emergency of the spread of SARS-CoV-2, but it could count on the knowledge of what happened in Italy, so it avoided the initial uncertainties in the management of the contagion. In fact, unlike Italy, it carried out widespread analysis on large areas of the territory where outbreaks of infection were reported, managing to improve containment measures for the initial spread of SARS-CoV-2. These measures have been very similar to those of Italy. Additionally, the dates for the reopening of the national borders coincide. Moreover, in Germany, as well as in Italy, it is obligatory to maintain physical distance and use face masks in closed spaces.

### Sweden

Sweden was one of the last European countries to recognize the presence of the COVID-19 coronavirus on its territory. Officially, the first case was ascertained approximately one month after the first case in Italy. The response of the Swedish authorities to the pandemic has been different from the response of other European nations. Sweden has imposed very few restrictions and has kept much of the economic activities open. This choice is dictated by the Swedish Constitution, which legally protects people's freedom of movement, thus preventing impositions in peacetime. Instead, the Public Health Authority provided guidelines of behavior to be considered on a voluntary basis. However, certain measures have been put into place by passing new laws, such as the closure of higher-order schools and universities, and the restriction of gatherings larger than 50 people. The use of face masks in Sweden is very limited, which has led to our decision of not including the use of face masks in the simulations for Sweden.

### Brazil

Brazil was the first South American country to have a recognized case of COVID-19 virus infection. The reactions of the Brazilian authorities in the face of the spread of SARS-CoV-2 have been uncoordinated and conflicting. Whereas health authorities were pushing to adopt policies measures similar those seen in Italy and Germany, the central government did not implement such measures. In addition to the lack of a strong policy response to the pandemic, the spread of SARS-CoV-2 was also favored by the living conditions of millions of Brazilians who reside in the suburbs of major cities, where it is challenging to adhere to required standards of hygiene and public health.

## Variables considered in the model

Transmission of SARS-CoV-2. The main phase on which the model is based is the transmission of SARS-CoV-2. Starting from a few infected individuals, SARS-CoV-2 spreads to other individuals who come into contact with those infected. Not all individuals who come into contact with those infected get infected; an algorithm takes into account both the transmissibility and the proximity of multiple infected individuals. If the governments adopt one or several preventive measures (e.g., the use of face masks and physical distancing), then transmissibility decreases for the duration of the measures. The decrease subsequently depends on the type and intensity of the adopted measure (i.e., obligation or advice).

Propagation speed. In addition to the degree of transmissibility and the proximity of multiple infected people, the speed with which the infection spreads, the propagation speed, further depends on the speed with which the individuals move. This speed is also considered as a productivity index and influences the GDP. Policy decisions aiming to restrict mobility will not only decrease the probability of SARS-CoV-2 transmission, but will also decrease the productivity of the firms which, in turn, has consequent effects on the national economy.

### Population-specific variables

Age distribution. In accordance with national population statistics (see Table 1), the population of each country was divided into two main categories, individuals aged under and over 65 years of age, respectively. This division was made for two reasons. First, from an economic perspective, those over the age of 65 do not contribute to the formation of the GDP in the same way as those under the age of 65. Consequently, movement speed of those over 65 is reduced compared to those under 65. Second, from a medical perspective, older individuals have a higher probability of comorbidities, which in turn may increase mortality rates compared to those who are younger.

### Country-specific variables

Country-specific policies. We analyze the effects of the political decisions of four countries that have had different approaches and outcomes: Italy was the first European state to face the emergency, Germany had a less rigid approach than Italy, but recorded a lower number of infections and deaths, Sweden had a "non-rigid" approach and the taken measures were provided as advice, and Brazil did not have a central role in the political decisions affecting the whole country.

Policy implementation timeline. The model follows the timeline of each considered country, replicating the taken measures both in the lockdowns and in the reopening, and records the effects of those measures over time.

Recognition of SARS-CoV-2. We further assume that SARS-CoV-2 is not immediately recognized from the first reported case in each country. Instead, SARS-CoV-2 has time to propagate. This is taken into account in the model, which thus records each country's ability to recognize the onset of the pandemic. The virus recognition periods may therefore vary depending on the respective country.

Availability of ICU beds. Hospitalizations start after the first case has been diagnosed. In the model, the number of intensive care unit (ICU) beds is assumed to be limited. We define an index that establishes the maximum number of available beds to treat seriously ill patients (based on the data provided by government bodies). When the maximum number of ICU beds is reached, the patients can no longer be hospitalized until a bed becomes available. This, in turn, causes an increase in the probability of death among seriously ill patients. The model shows the bed occupancy rate (percent). It is possible to remove this limitation on beds, in which case the model shows the number of beds that would have been needed to meet the demands during the evolution of the pandemic.

Productivity. The political decisions to reduce the mobility of people have caused large and negative economic repercussions due to the effects on production and commercial activities. The model analyses the trend of the economy during the various lockdown phases and displays the final results for each analyzed country with respect to the initial data.

### Virus-specific variables

Incubation period. The model considers that there is an incubation period for SARS-CoV-2. Based on the data provided by the European Center for Disease Prevention and Control (ECDC), which indicate an incubation period that varies from 2 to 14 days [45], we differentiate the incubation periods of our population according to age. For individuals under the age of 65 years, we set the incubation period according to a normal distribution with mean 7 and standard deviation 2 ($I_{Y} \sim N(7,4)$), while for those who are over the age of 65, the incubation period is defined according to a normal distribution with mean 3 and standard deviation 1 ($I_{O} \sim N(3,1)$). The reason for this differentiation between older and young people for the duration of the virus incubation is due to the fact, now recognized by the WHO and by all the national health organizations, that older people have a higher probability of occurrence of a serious illness following the infection, with also a consequent manifestation of the symptoms (such as fever and cough) within a short period [46]. On the contrary, most young people who contract the disease are less likely to have a serious infection and, often, they are asymptomatic (14.3% of asymptomatic versus 6.3% for over 65s) [46]. During the incubation period, cases have the ability to infect other people. This capacity grows as the virus develops, until reaching a peak at the end of the incubation period.

Asymptomatic cases. The model takes into account that not all cases are symptomatic. The absence of symptoms makes these individuals more difficult to identify, but they will also have a lower infectious capacity than symptomatic cases. Indeed, experts from international health institutions (such as the WHO) believe that the transmission of the virus by asymptomatic cases does not contribute much to its general spread, because in many cases the viral load is low and the absence of symptoms such as cough and cold limits its diffusion [47].

Non-contagious asymptomatic cases. The model takes into account non-contagious asymptomatic cases. i.e., a small proportion of individuals who, following infection, develop antibodies without presenting symptoms and never become carriers of SARS-CoV-2.

Severity of COVID-19. Not all the infected individuals are hospitalized as serious cases as most of them have milder symptoms that can be treated without requiring intensive care (the data are provided by the public health bodies, see Table 1). Given the relatively small number of agents on which we are running the simulation, the discrimination of the severity of the disease was conducted on two levels only: seriously ill people in need of intensive care, and non-critically ill people which include both those who are hospitalized with milder symptoms and those placed in home isolation. The hospitalized patients will no longer have the possibility of infecting other people, while those in home isolation will limit the spread of disease to family members only.

Recovery and case fatality rate. Seriously ill cases can recover or die (the statistical data for the defined indexes are provided by the respective countries). The recovery probability will be higher for those who are under the age of 65 years than for those who are older. On the contrary, those who are over the age of 65 years will have a higher case fatality rate compared to those who are under the age of 65 years. In the model, those who recover from a serious infection are considered to have obtained life-long immunity to the SARS-CoV-2. Even people not admitted to intensive care, i.e., less severe cases, can recover or die. However, their chances of recovery are higher than those of hospitalized patients. Again, the probability of recovery is higher for people under the age of 65 years. Irrespectively, those who have recovered from a less severe infection are to be considered immune to the virus for a certain period, but will not have lifelong immunity.

Basic and effective reproduction numbers. The model calculates and records the daily values of R_0_ (basic reproduction number) and R_E_ (effective reproduction number).

Herd immunity. At each period, the threshold value necessary to be reached for obtaining herd immunity in the population is calculated. This value depends on R_0_. When the number of infections exceeds the immunity herd peaks, it could be considered that herd immunity has been reached.

### Policy-specific parameters

Use of face masks. The use of face masks lowers the probability of contagion by 14.3% [48]. The model takes into account the governmental requirements for the use of face masks, and changes the virus transmissibility parameters accordingly. Italy and Germany have this value set at 14.3%, due to the mandatory use of face masks. For Brazil the value is halved as the use of face masks is not mandated, while for Sweden we set this value at zero, since the use of face masks is not recommended by the public health authority.

Implementation of social distancing rules. Respecting physical distance lowers the probability of infection by 10.2% [48]. Again, the model takes into account governmental requirements and changes the transmissibility parameters of the virus accordingly. For Italy, Germany and Sweden this value is considered at 10.2% due to mandatory social distance. For Brazil, the value has been halved.

Reopening of national borders. The model provides for the possibility to reopen national borders to other countries when the situation returns under control. This restores the free movement of people. Despite any implemented controls, there will be a small percentage of travelers who will be carriers of the virus, with the possibility of re-introducing cases into the respective country. These changes are recorded in the contagion curves.

## References

45. Linton N M, Kobayashi T, Yang Y et al. Incubation period and other epidemiological characteristics of 2019 novel coronavirus infections with right truncation: a statistical analysis of publicly available case data. J Clin Med 2020; 9: E538–9. DOI: [10.3390/jcm9020538](https://doi.org/10.3390/jcm9020538)  PMID: 32079150

46. De Sanctis V, Ruggiero L, Soliman A T, Daar S, Di Maio S, Kattamis C. Coronavirus disease 2019 (COVID-19) in adolescents: An update on current clinical and diagnostic characteristics. Acta Biomed 2020; 91: 184–194 DOI: 10.23750/abm.v91i2.9543. PMID: 32420943

47. Gao M., Yang L, Chen X et al. A study on infectivity of asymptomatic SARS‐CoV‐2 carriers. Respir Med 2020; 169: 106026. [Elsevier Public Health Emergency Collection](https://www.ncbi.nlm.nih.gov/pmc/?term=Elsevier%20Public%20Health%20Emergency%20Collection%5bfilter%5d) PMC7219423. DOI: [10.1016/j.rmed.2020.106026](https://doi.org/10.1016/j.rmed.2020.106026) . PMID: 32513410

48. Chu D K, Akl E A, Duda S, Solo K, Yaacoub S, Schünemann H J. Physical distancing, face masks, and eye protection to prevent person to person transmission of SARS-CoV-2 and COVID-19: a systematic review and metaanalysis. Lancet 2020; 395: 1973–87. DOI: [10.1016/S0140-6736(20)31142-9](https://doi.org/10.1016/s0140-6736(20)31142-9). PMID: 32497510
